# Prognosis for Hospitalized Patients with Systemic Lupus Erythematosus in China: 5-Year Update of the Jiangsu Cohort

**DOI:** 10.1371/journal.pone.0168619

**Published:** 2016-12-28

**Authors:** Xuebing Feng, Wenyou Pan, Lin Liu, Min Wu, Fuwan Ding, Huaixia Hu, Xiang Ding, Hua Wei, Yaohong Zou, Xian Qian, Meimei Wang, Jian Wu, Juan Tao, Jun Tan, Zhanyun Da, Miaojia Zhang, Jing Li, Lingyun Sun, On behalf of Jiangsu Lupus Collaborative Group

**Affiliations:** 1 Department of Rheumatology and Immunology, the Affiliated Drum Tower Hospital of Nanjing University Medical School, Nanjing, China; 2 Department of Rheumatology, Huaian First People’s Hospital, Huaian, China; 3 Department of Rheumatology, Xuzhou Central Hospital, Xuzhou, China; 4 Department of Rheumatology, the Third Affiliated Hospital of Soochow University, Changzhou, China; 5 Department of Endocrinology, Yancheng Third People's Hospital, Yancheng, China; 6 Department of Rheumatology, Lianyungang Second People's Hospital, Lianyungang, China; 7 Department of Rheumatology, Lianyungang First People's Hospital, Lianyungang, China; 8 Department of Rheumatology, Northern Jiangsu People's Hospital, Yangzhou, China; 9 Department of Rheumatology, Wuxi People's Hospital, Wuxi, China; 10 Department of Rheumatology, Jiangsu Province Hospital of TCM, Nanjing, China; 11 Department of Rheumatology, Southeast University Zhongda Hospital, Nanjing, China; 12 Department of Rheumatology, the First Affiliated Hospital of Soochow University, Suzhou, China; 13 Department of Rheumatology, Wuxi TCM Hospital, Wuxi, China; 14 Department of Rheumatology, Zhenjiang First People’s Hospital, Zhenjiang, China; 15 Department of Rheumatology, Affiliated Hospital of Nantong University, Nantong, China; 16 Department of Rheumatology, Jiangsu Province Hospital, Nanjing, China; 17 Department of Rheumatology, Affiliated Hospital of Jiangsu University, Jiangsu, China; JAPAN

## Abstract

**Objective:**

To identify early signs associated with poor prognosis in Chinese patients with systemic lupus erythematosus (SLE) through a large population-based follow-up study.

**Methods:**

Medical records of > 2,500 SLE patients that first hospitalized between 1999–2009 were collected from 26 centers across Jiangsu province, China, and entered into a database. These patients were followed-up for 5 to 15 years, and those remained contact and had known survival status in 2015 were assessed for the association of factors presented at the initial hospitalization with mortality at two time points (≤1year and > 1year). The independency of mortality factors was evaluated using multivariate Cox regression analysis.

**Results:**

Among 1,372 patients we assessed, 92.3% were women and 17.2% were deceased in 2015. The main causes of death were infection (30.1%), neuropsychiatric impairment (14.8%), renal failure (14.4%) and cardiopulmonary involvement (8.5%). Hazard ratios (HR) of independent predictors for mortality (≤1year and > 1year, respectively) included hospital presentation of neuropsychiatric involvement (2.03 and 1.91), cardiopulmonary involvement (1.94 and 1.61) and increased serum creatinine (2.52 and 2.58). Patients older than 45 years and with disease durations more than 2 years at admission had unfavorable short-term outcome (HR 1.76 and 1.79), while the presence of anti-dsDNA and anti-Sm antibodies indicated diverse prognosis after 1 year (HR 1.60 and 0.45). Treatment with cyclophosphamide was beneficial for patient’s first-year outcome (HR 0.50), and anti-malarial drugs significantly reduced the risk of mortality over different time points (HR 0.48 and 0.54). SLEDAI score, proteinuria or hypocomplementemia was not independently associated with the outcome in this cohort.

**Conclusion:**

SLE patients presented with vital organ damages rather than active disease at initial hospitalization are likely to have a poor outcome, especially for those with neuropsychiatric, cardiopulmonary involvements and renal insufficiency. Early and effective intervention with the use of anti-malarial drugs may decrease mortality.

## Introduction

Systemic lupus erythematosus (SLE) is a chronic, multifaceted autoimmune disease that causes a wide variety of signs and symptoms. Although the 5-year survival rate has dramatically improved in the past 50 years, from 50% in the 1950s to over 90% since the 1990s, likely due to early diagnosis and intervention followed by a better understanding of the disease characteristics, SLE patients still have risk of death that is two to five times higher than the general population[1]. As a highly heterogeneous disease, SLE may lead to varied disease severity and outcomes among individuals. To improve overall survival, early identification of subgroups with poor outcome in SLE patients is of crucial importance.

Different from those in western countries where cardiovascular involvement and tumors became the leading causes of death, Asian patients with SLE were observed to have more deaths due to infections and active disease [2]. To evaluate the survival pattern of Chinese SLE patients and its outcome according to the earliest clinical and laboratory presentations as well as treatments, we have carried out a long-term project to follow-up the patients hospitalized during the 1999–2009 decade in Jiangsu province, China, since 2010 [3]. In 2015, the survival status of these patients was updated and risk factors related to mortality at two time points (≤1year and > 1year) were analyzed.

## Methods

### Study design

To delineate the prognosis of SLE patients in China, a follow-up study has been conducted by the Lupus Collaborative Group under the supervisor of Jiangsu Rheumatology Association. With the help of Cinkate Corp, a website (formerly http://lupus.cinkate.com.cn:2222/ and now http://sys.91sqs.com/sle) has been built to collect medical records of hospitalized SLE patients from January 1, 1999 to December 31, 2009 in Jiangsu Province, China, since 2010. All patients fulfilled at least 4 of the revised and/or updated American College of Rheumatology criteria for classification of SLE [4,5]. Up to 2015, data of over 2,500 cases were documented. Among which, patients who had ever recorded first admissions during the 1999–2009 decade were followed and checked for their survival status between April and June 2015, and those lost to contact were excluded from this study.

### Data collection and definition

Totally 26 centers across Jiangsu province were participated and 1,372 SLE patients were enrolled, in which 236 were deceased and 1,136 remained alive. The patients’ clinical data on first admission, including gender, age, disease duration, diagnostic time, disease activity and damage, organ involvements, laboratory findings, treatments, follow-up time and cause of death (if any) were extracted from the database.

Disease activity was calculated according to the SLE Disease Activity Index (SLEDAI) score [6] and organ damage was determined by the Systemic Lupus International Collaborating Clinics (SLICC)/American College of Rheumatology (ACR) Damage Index (SDI) [7]. Specific organ involvements were defined as having one of the following manifestations: 1) ***Mucocutaneous***: skin eruption, mucosal ulceration, cutaneous vasculitis, alopecia, digital infarcts, periungual erythema, angioedema or panniculitis; 2) ***Musculoskeletal***: arthritis/ arthralgia, myositis/ myalgia; 3) ***Neuropsychiatric***: headache, epilepsy, cerebral vasculitis, cerebrovascular disease, demyelination syndrome, myelopathy, aseptic meningitis, cerebellar ataxia, mononeuropathy, polyneuropathy, psychosis, acute confusional state, mood disorder (depression/mania); 4) ***Cardiopulmonary***: serositis, myocarditis, interstitial lung disease, pulmonary arterial hypertension, pulmonary hemorrhage/vasculitis, cardiac failure, arrhythmia, valvular dysfunction; 5) ***Renal***: proteinuria, hematuria, active urinary sediment, increased serum creatinine or abnormal glomerular filtration rate (GFR), hypertension (renal related), biopsy-proved lupus nephritis; 6) ***Gastrointestinal***: peritonitis, ascites, malabsorption, hepatitis/abnormal liver function, mesenteric vasculitis, protein-losing enteropathy, lupus gastroenteritis, pancreatitis; 7) ***Hematological***: hemolytic anemia, leukopenia, thrombocytopenia. The data were collected anonymously and none of the authors had access to information that could identify individual participants after data collection.

Normal values for laboratory tests were as follows: white blood cells ≥ 4 ×10^9^/L, hemoglobin ≥ 110 g/L (female) or 120 g/L (male), platelets ≥ 100 ×10^9^/L, alanine aminotransferase (ALT) ≤ 50 IU/L, aspartate aminotransferase (AST) ≤ 50 IU/L, serum albumin ≥ 35 g/L, blood urea nitrogen (BUN) ≤ 7.5mmol/L, serum creatinine ≤ 133 μmol/L, complement C3 ≥ 0.8 g/L, C4 ≥ 0.2 g/L, anti-nuclear antibody (ANA) ≤ 1: 40, anti-dsDNA antibody negative, anti-Sm antibody negative, anti-cardiolipin antibody < 12 U/ml or negative, rheumatoid factor (RF) < 20 IU/ml, urine protein< 0.5 g/24hr or less than 2+. All the antibodies tested were IgG type and the negativity was defined according to the standard in each hospital.

### Statistics

Data were analyzed using SPSS 21.0 software, and the Kaplan-Meier survival plot was drawn by Graphpad Prism 6.0. Values were expressed as number (percentage) or median (quartiles). Comparisons between two groups were performed by the chi-square test. To determine the risk factors for mortality at two time points (defined as died ≤ 1year and > 1 year after first hospitalization) in SLE patients, univariate analysis using the Cox proportional hazard model was conducted. Variables significant (p<0.05) in univariate analysis were then included in the multivariate model, with missing data treated as normal values. Results were reported as hazard ratios (HR) with 95% confidence intervals (CI), and p < 0.05 was considered statistically significant.

## Results

### Patient characteristics

**Fig 1:**
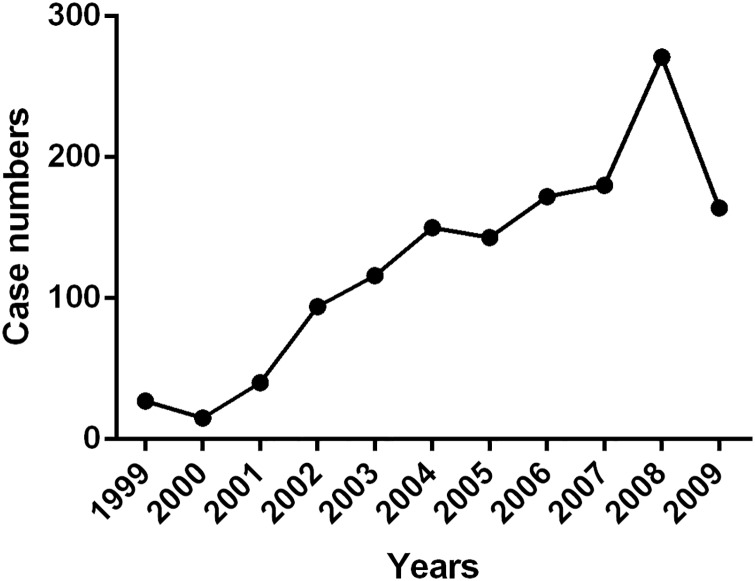
Number of SLE patients recruited each calendar year (1999–2009).

As shown in Fig 1, 15 to 271 patients were recruited each calendar year between 1999 and 2009. Among the 1,372 SLE cases, 92.3% were female (Table 1). The age of hospital admission was 34.3 (24.9, 42.2) years, with an average duration of 1.1 (0.2, 4.7) years after disease onset. SLEDAI scores were 13 (9, 19) on admission and 4 (0, 9) at discharge. 12.5% of patients had SDI ≥ 1 and 5.0% had SDI ≥ 2, in which most of them was steroids irrelevant (S1 Table). 12.6% of patients had comorbidities at the time of admission, in which Sjögren's syndrome was the most common accompanied autoimmune disease (24 cases), while diabetes (65 cases), infection (37 cases) and hypertension (31 cases) accounted for the most frequent non-autoimmune disorders.

**Table 1:** Demographics of 1,372 SLE patients.

Variables	Values ^a^
Age of admission, years	34.3 (24.9, 42.2)
Gender (female)	1,266 (92.3%)
Disease duration, years	1.13 (0.20, 4.67)
Time between onset to diagnosis, years	0.25 (0.07, 1.45)
SLEDAI score on admission	13 (9, 19)
SDI ≥ 1 on admission	171 (12.5%)
Comorbidities	173 (12.6%)
Organ involvements	
Mucocutaneous	914 (66.6%)
Musculoskeletal	744 (54.2%)
Neuropsychiatric	92 (6.7%)
Cardiopulmonary	282 (20.6%)
Gastrointestinal	314 (22.9%)
Renal	701 (51.1%)
Hematologic	617 (45.0%)
Antibody profiles	
ANA positive	1,159 (92.4%)
Anti-dsDNA positive	641 (52.8%)
Anti-Sm positve	403 (33.6%)
Anti-cardiolipin positive	146 (29.5%)
RF positive	243 (28.7%)
Hypocomplementemia	1,064 (89.3%)
Treatments	
Steroids	1,264 (92.1%)
Anti- malarial drugs	562 (41.0%)
Cyclophosphamide	582 (42.4%)
Other immunosuppressives	233 (17.0%)

^a^ Values were presented by number (percentage) or median (quartiles). Percentages were calculated as the positive numbers divided by total numbers available in each category.

The incidences of mucocutaneous, musculoskeletal, neuropsychiatric, cardiopulmonary, renal, gastrointestinal and hematological involvements were 66.6%, 54.2%, 6.7%, 20.6%, 51.1%, 22.9% and 45.0% respectively. Positive rates for ANA, anti-dsDNA, anti-Sm, anti-cardiolipin and RF were 92.4%, 52.8%, 33.6%, 29.5% and 28.7%, and hypocomplementemia (defined as having decreased levels of complement C3 or C4) was displayed in 89.3% of the patients. In this cohort, 92.1% received steroids, 41.0% received anti- malarial drugs (chloroquine or hydroxychloroquine), 42.4% received cyclophosphamide and 17.0% received other immunosuppressives (including methotrexate, azathioprine, leflunomide, mycophenolate mofetil, cyclosporine, tacrolimus and tripterygium wilfordii multiglycosides [also known as thunder god vine]) during hospitalization.

### Cause of death

**Fig 2:**
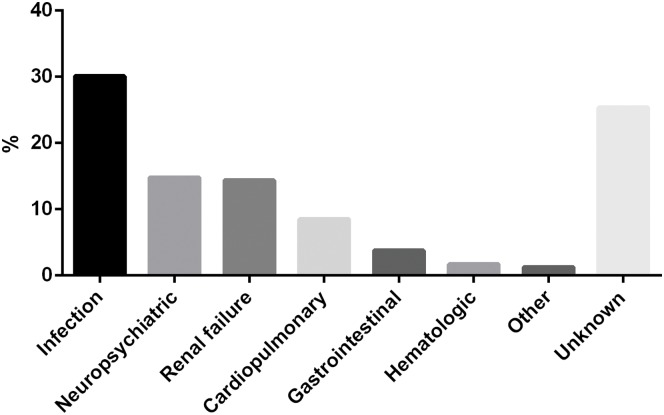
Causes of death among the 236 deceased patients. The most often seen cause of death was infection (30.1%), followed by neuropsychiatric impairments (14.8%), renal failure (14.4%) and cardiopulmonary involvements (8.5%). Ill-defined causes of death, classified as unknown here, represented 25.4% of the total deaths.

236 patients died before the 2015 visit. Of which, 176 (74.6%) had available cause of death data other than cardiopulmonary failure or multiple organ failure. The primary cause of death was summarized in Fig 2. Infection remained the leading cause of death (30.1%), in which nearly 2/3 were pulmonary infection. 14.8% of the patients died of neuropsychiatric impairments, including neuropsychiatric lupus (n = 25), intracranial hemorrhage (n = 7) and cerebral hernia (n = 3), which was nearly the same as that of renal failure (14.4%). Twenty died of cardiopulmonary involvements, including pulmonary arterial hypertension (n = 10), pulmonary involvement (n = 5), myocardial damage (n = 2), sudden death (n = 2) and aortic dissection (n = 1) (S2 Table). Nine died of gastrointestinal complications, including liver failure (n = 4), gastrointestinal bleeding (n = 3), digestive tract perforation (n = 1) and acute pancreatitis (n = 1). Other causes of death included hematologic disorder (n = 4), femoral head osteonecrosis, suicide and tumor.

**Fig 3:**
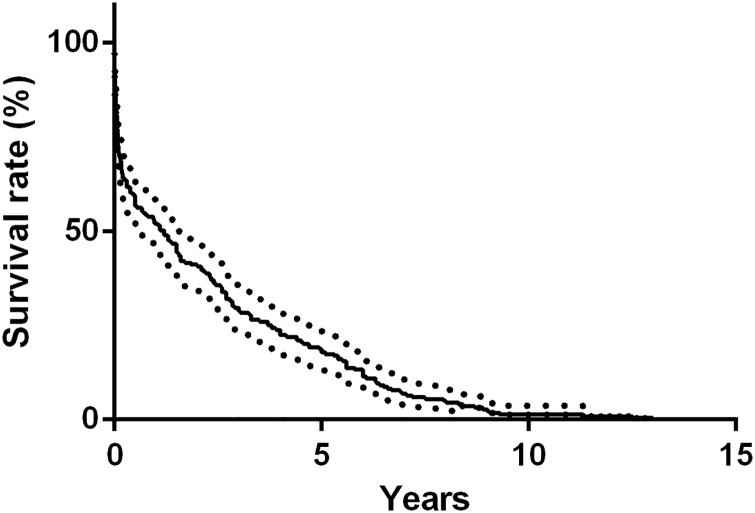
Kaplan-Meier estimated survival curves for deceased cases with known time of death (n = 219). Nearly a half (47.9%) of the patients died within 1 year after first hospitalization.

**Fig 4:**
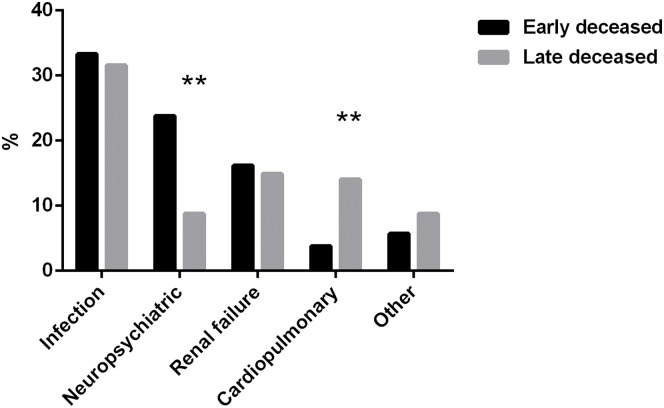
Different causes of death between early and late deceased patients. Compared with those died within 1 year after first hospitalization, patients died 1 year later had more cardiopulmonary related deaths (14.0% vs. 3.8%) but less neuropsychiatric related deaths (8.8% vs. 23.8%).

For patients with known time of death, when stratified by the time of first admission, 105 occurred within 1 year (47.9%), 75 occurred between 1 to 5 years (34.2%) and only 39 occurred 5 year later (17.8%) (Fig 3). Compared with those died early, patients died 1 year later had more deaths due to cardiopulmonary involvements (14.0% vs. 3.8%, p < 0.01) but fewer due to neuropsychiatric impairments (8.8% vs. 23.8%, p < 0.01) (Fig 4).

### Factors related to mortality within 1 year

The Cox proportional hazard model was used for the detection of risk factors contributing to first-year mortality in SLE patients by analyzing the data of cases died within 1 year after the first admission and those remained alive. As shown in Table 2, in univariate analysis, male gender, older age at admission, long disease duration, high SLEDAI scores both in admission and at discharge, organ involvements (neuropsychiatric, cardiopulmonary, gastrointestinal, renal and hematologic), anemia, thrombocytopenia, elevated transaminases, hypoalbuminemia, proteinuria and increased blood urea nitrogen or serum creatinine were associated with worse prognosis, while the application of anti-malarial drugs or cyclophosphamide was associated with a better outcome. Most of the patients had hypocomplementemia in this cohort, yet it did not constitute a risk factor for death. Multivariate analysis revealed that older age at admission (HR 1.76, p < 0.01), disease duration > 2 years (HR 1.79, p < 0.01), neuropsychiatric involvement (HR 2.03, p < 0.05), cardiopulmonary involvement (HR 1.94, p < 0.01), anemia (HR 1.76, p < 0.05), increased blood urea nitrogen (HR 2.10, p < 0.01) and increased serum creatinine (HR 2.52, p = 0.001) were independent predictors of mortality. Meanwhile, treatments with either anti-malarial drugs (HR 0.48, p < 0.01) or cyclophosphamide (HR 0.50, p = 0.001) were shown to be independent protective factors.

**Table 2:** Factors associated with first-year mortality (Cox proportional hazards model).

Factors		Univariate			Multivariate		
		HR	95%CI	p	HR	95%CI	p
Gender (male)		2.01	1.16–3.46	0.013	1.22	0.68–2.19	>0.05
Older age ^a^ at first admission		2.09	1.43–3.04	0.000	1.76	1.19–2.62	0.005
Disease duration > 2 years		2.11	1.42–3.13	0.000	1.79	1.17–2.73	0.007
Time of diagnosis > 1year		1.18	0.75–1.88	>0.05			
SLEDAI > 15 in admission		2.16	1.46–3.18	0.000	1.19	0.73–1.92	>0.05
SLEDAI > 10 at discharge		2.29	1.53–3.43	0.000	1.19	0.74–1.90	>0.05
SDI ≥ 2 in admission		1.42	0.66–3.05	>0.05			
Organ involvements	Mucocutaneous	0.74	0.50–1.10	>0.05			
	Musculoskeletal	0.77	0.52–1.13	>0.05			
	Neuropsychiatric	3.48	2.10–5.78	0.000	2.03	1.16–3.56	0.013
	Cardiopulmonary	3.42	2.33–5.03	0.000	1.94	1.27–2.98	0.002
	Gastrointestinal	2.44	1.65–3.60	0.000	0.81	0.19–3.40	>0.05
	Renal	1.82	1.22–2.71	0.003	0.47	0.19–1.17	>0.05
	Hematologic	2.58	1.72–3.88	0.000	0.90	0.45–1.79	>0.05
Lab tests	Leukopenia	1.14	0.77–1.68	>0.05			
	Anemia	3.55	2.11–5.97	0.000	1.76	1.03–3.01	0.040
	Thrombocytopenia	4.16	2.80–6.18	0.000	1.81	0.93–3.53	>0.05
	Elevated transaminases	2.61	1.75–3.89	0.000	2.47	0.58–10.6	>0.05
	Hypoalbuminemia	3.10	1.92–5.01	0.000	1.50	0.90–2.52	>0.05
	Increased blood urea nitrogen	4.60	3.11–6.80	0.000	2.10	1.29–3.42	0.003
	Increased serum creatinine	6.09	3.91–9.49	0.000	2.52	1.44–4.41	0.001
	Proteinuria	1.97	1.31–2.96	0.001	1.58	0.67–3.74	>0.05
	Decreased complement C3	0.90	0.56–1.44	>0.05			
	Decreased complement C4	1.08	0.62–1.88	>0.05			
	Positive anti-dsDNA	1.28	0.85–1.95	>0.05			
	Positive anti-Sm	0.76	0.48–1.21	>0.05			
	Positive RF	1.19	0.72–1.98	>0.05			
Treatments	Steroids	0.95	0.48–1.88	>0.05			
	Anti-malarial drugs	0.37	0.23–0.58	0.000	0.48	0.30–0.78	0.003
	Cyclophosphamide	0.60	0.40–0.90	0.014	0.50	0.32–0.76	0.001
	Other ISA ^b^	1.01	0.61–1.68	>0.05			

^a^ > 45 years.

^b^ including methotrexate, azathioprine, leflunomide, mycophenolate mofetil, cyclosporine, tacrolimus and tripterygium wilfordii multiglycosides

### Factors related to mortality over 1 year

Risk factors related to mortality over 1 year in SLE patients were analyzed by Cox proportional hazards regression using the data of patients alive and died 1 year later after the first admission. Univariate analysis showed that long disease duration, high SLEDAI score in admission, neuropsychiatric, cardiopulmonary and renal involvements, anemia, hypoalbuminemia, proteinuria, increased blood urea nitrogen or serum creatinine and positive anti-dsDNA were associated with worse prognosis, while positive anti-Sm and the application of anti-malarial drugs or cyclophosphamide were linked to a better outcome. By multivariate analysis, only neuropsychiatric involvement (HR 1.91, p < 0.05), cardiopulmonary involvement (HR 1.61, p < 0.05), increased serum creatinine (HR 2.58, p = 0.001) and positive anti-dsDNA (HR 1.60, p < 0.05) were independent predictors of mortality. Meanwhile, having positive anti-Sm (HR 0.45, p = 0.001) and being treated with anti-malarial drugs (HR 0.54, p < 0.01) were beneficial to the patient’s long-term survival (Table 3).

**Table 3:** Factors associated with mortality over 1 year (Cox proportional hazards model).

Factors		Univariate			Multivariate		
		HR	95%CI	p	HR	95%CI	p
Gender (male)		0.99	0.48–2.04	>0.05			
Older age at first admission		1.26	0.86–1.85	>0.05			
Disease duration > 2 years		1.49	1.03–2.15	0.035	1.45	0.98–2.16	>0.05
Time of diagnosis > 1year		0.86	0.55–1.34	>0.05			
SLEDAI > 15 in admission		1.53	1.06–2.21	0.024	1.05	0.69–1.61	>0.05
SLEDAI > 10 at discharge		1.40	0.91–2.16	>0.05			
SDI ≥ 2 in admission		0.93	0.38–2.29	>0.05			
Organ involvements	Mucocutaneous	0.97	0.66–1.43	>0.05			
	Musculoskeletal	0.81	0.56–1.17	>0.05			
	Neuropsychiatric	2.17	1.22–3.86	0.009	1.91	1.03–3.52	0.039
	Cardiopulmonary	2.12	1.43–3.15	0.000	1.61	1.06–2.46	0.026
	Gastrointestinal	1.31	0.86–1.98	>0.05			
	Renal	1.79	1.22–2.62	0.003	0.53	0.20–1.41	>0.05
	Hematologic	1.12	0.78–1.62	>0.05			
Lab tests	Leukopenia	0.94	0.65–1.37	>0.05			
	Anemia	2.15	1.40–3.30	0.000	1.46	0.93–2.31	>0.05
	Thrombocytopenia	1.45	0.97–2.15	>0.05			
	Elevated transaminases	1.44	0.94–2.20	>0.05			
	Hypoalbuminemia	2.12	1.40–3.22	0.000	1.41	0.90–2.20	>0.05
	Increased blood urea nitrogen	2.55	1.72–3.77	0.000	1.24	0.76–2.03	>0.05
	Increased serum creatinine	4.16	2.60–6.66	0.000	2.58	1.44–4.61	0.001
	Proteinuria	1.80	1.23–2.63	0.002	2.00	0.79–5.09	>0.05
	Decreased complement C3	1.00	0.63–1.59	>0.05			
	Decreased complement C4	1.05	0.62–1.77	>0.05			
	Positive anti-dsDNA	1.67	1.11–2.49	0.013	1.60	1.09–2.37	0.017
	Positive anti-Sm	0.46	0.28–0.76	0.002	0.45	0.28–0.74	0.001
	Positive RF	0.98	0.55–1.75	>0.05			
Treatments	Steroids	1.45	0.64–3.31	>0.05			
	Anti-malarial drugs	0.49	0.32–0.74	0.001	0.54	0.35–0.82	0.004
	Cyclophosphamide	0.87	0.60–1.26	>0.05			
	Other ISA	1.20	0.75–1.90	>0.05			

### Treatments for patients with vital organ involvements

**Fig 5:**
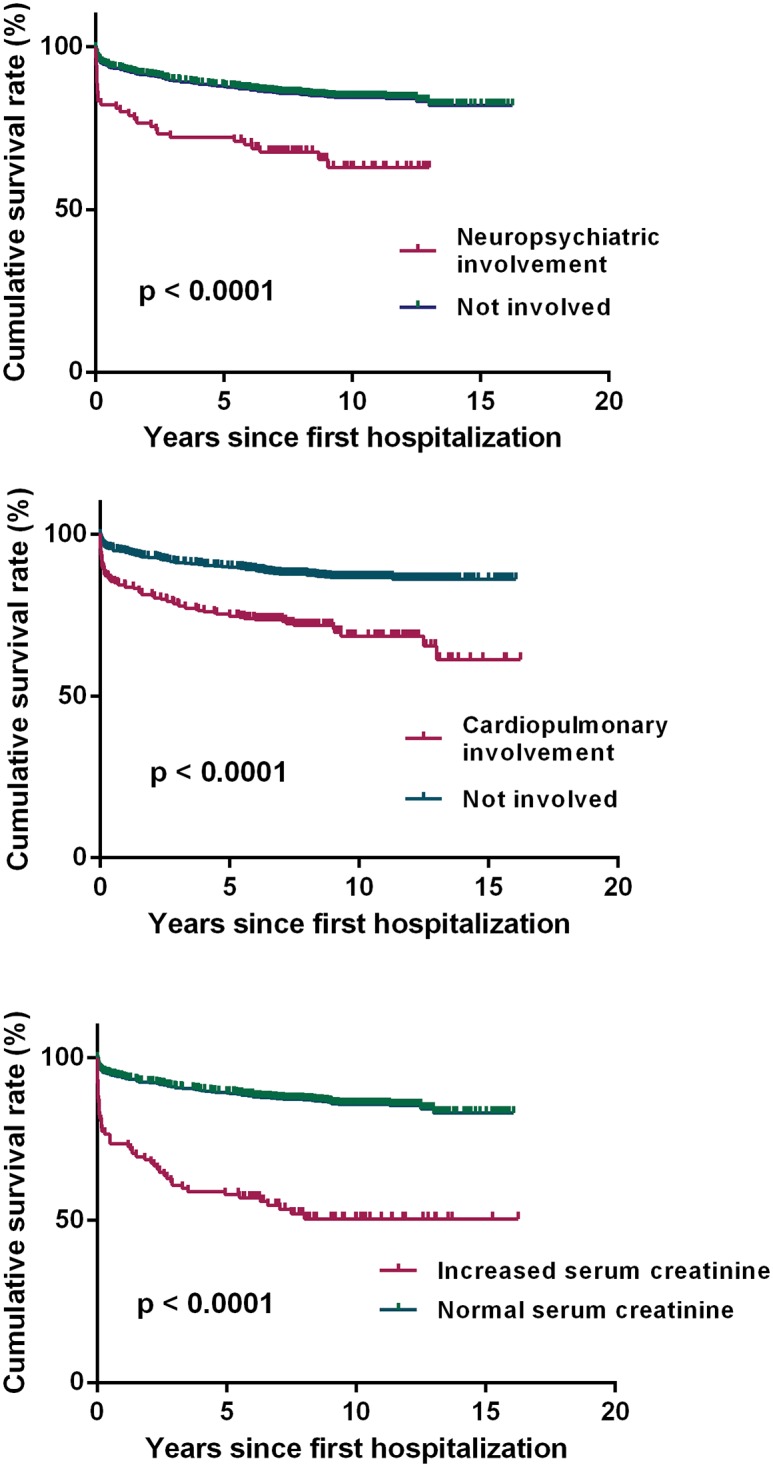
Cumulative survival rates by individual variables. a) Kaplan-Meier survival estimates according to neuropsychiatric involvement. b) Kaplan-Meier survival estimates according to cardiopulmonary involvement. c) Kaplan-Meier survival estimates according to serum creatinine level.

**Fig 6:**
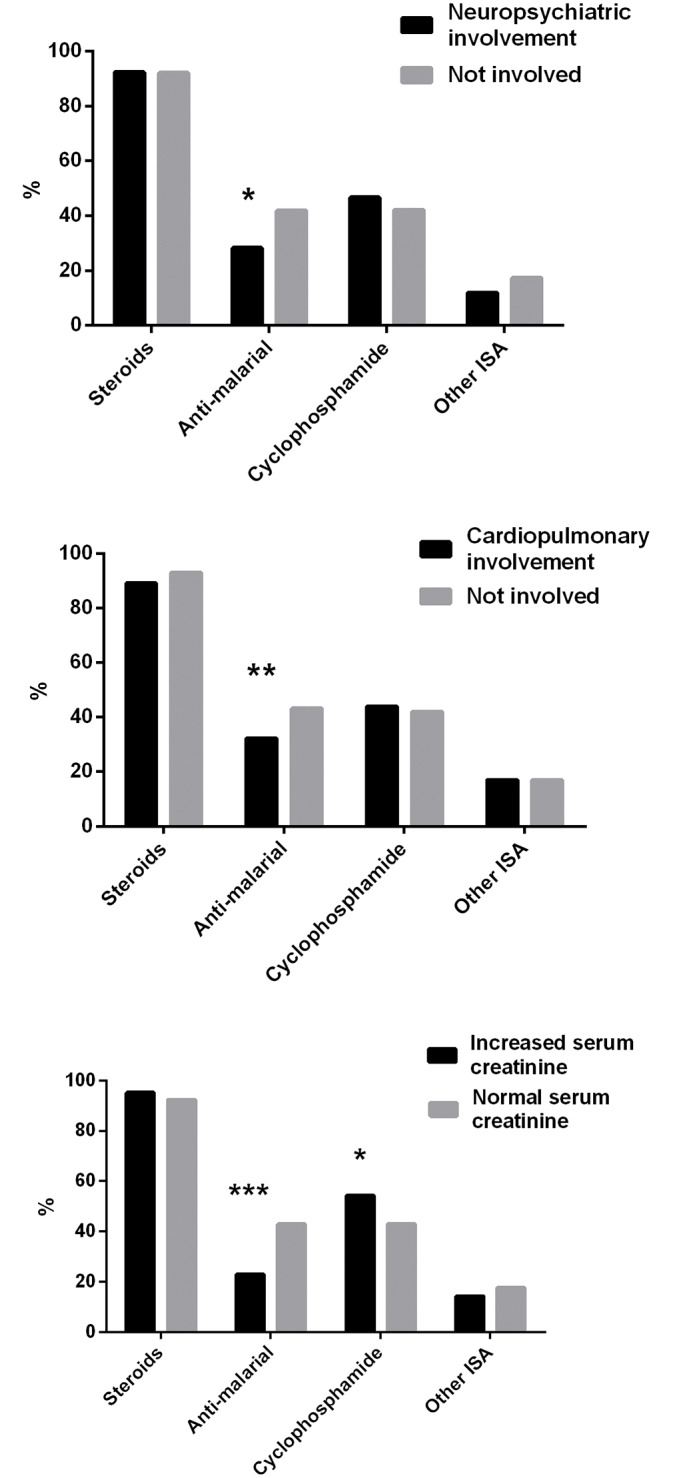
Major therapeutic option for SLE patients with various manifestations during the first hospitalization. a) For patients with or without neuropsychiatric involvement. b) For patients with or without cardiopulmonary involvement. c) For patients with or without increased serum creatinine level.

The ten year survival rates for patients with neuropsychiatric, cardiopulmonary involvements and increased serum creatinine were 62.9%, 68.6% and 50.5% respectively in this cohort (Fig 5). To explore whether treatment strategies were related to the outcome of these patients at the time of first admission, the prescription percentages of steroids, anti-malarial drugs, cyclophosphamide and other immunosuppressives were calculated. As shown in Fig 6, patients with neuropsychiatric or cardiopulmonary involvement had lower chances to take anti-malarial drugs, while patients with renal insufficiency were less likely to be treated with anti-malarial drugs but more likely to be treated cyclophosphamide.

## Discussion

To the best of our knowledge, this is the first long-term follow-up multicenter study for SLE patients from Jiangsu providence that has a ~80 million population s in China. Our team tracked down the survival status of hospitalized SLE patients from 26 centers in Jiangsu province for 5–15 years and attempted to reveal early signs pointed to poor prognosis. The data demonstrated that SLE patients with neuropsychiatric, cardiopulmonary involvements or abnormal serum creatinine level had increased mortality at two time points (≤1year and > 1year). Patients older than 45 years or with disease durations more than 2 years at admission were expected to have unfavorable first-year outcome, while the presence of autoantibodies, such as anti-dsDNA and anti-Sm, was of value in evaluating the prognosis after 1 year. Treatment with cyclophosphamide was beneficial for patient’s short-term outcome, and anti-malarial drugs significantly reduced the risk of mortality over different time periods.

In the present study, the majority deaths of SLE patients were resulted by infections or vital organ (especially neuropsychiatric, renal and cardiopulmonary) involvements due to the progression of lupus itself, which was quite different to those of the same period in western countries. In the United Kingdom, France, Finland and Italy, cardiovascular disease and malignancy have been claimed as the two leading causes of death [8–11]. The discrepancy could be partly attributed to different age of patients among the groups, as patients included in most of the other studies were much older than ours. To support this notion, it has been reported that elder patients were more often died with SLE as the non-underlying cause instead of the underlying cause [9,12]. In addition, patients in the Asia-Pacific region have been identified to possess more severe manifestations than Caucasians [13], which may also contribute to the high incidence of SLE related deaths in this group.

A bimodal distribution of mortality in SLE has long been proposed, with a first peak occurred within two years after diagnosis and second peak appeared five years later [14]. In this study, although nearly half of the deaths occurred in the first year after hospitalization, a hyperbolic pattern was displayed on the survival curve. Our data did prove a bimodal distribution of the causes of death in SLE, showing that more patients died of neuropsychiatric impairments during the early period and more died of cardiopulmonary involvements during the late period. Apart from these aspects, deaths caused by infections or renal failures remained steady over different periods.

Consistent with previous reports [14], over half of SLE patients had renal involvement during their first admission. However, according to our multivariate Cox regression analysis, lupus nephritis was not independently related to a poor outcome. A possible explanation is that renal involvements are a marker of severe disease and usually accompanied by the presence of other vital organ damage. Based on our and other studies [15,16], increased level of serum creatinine is more useful in predicting mortality than renal involvement or merely proteinuria. Similarly, because hypocomplementemia is often a harbinger of active renal disease, it would not be surprised to find out that decreased complement component was irrelevant to survival over different time periods. In line with our expectation 5 years ago [3], in this study, anti-Sm antibody was not relevant to short-term mortality, while patients with positive anti-Sm were more likely to survive one year later after the first hospitalization. As a specific marker for SLE diagnosis, positivity of anti-Sm could lead to early identification of patients at hospital admission and prompt treatment. These patients may not fare well within a short-term because of their active disease (S3 Table), but their mortality risk is reduced in the longer observation time.

Our data confirms that patients taken anti-malarial drugs had decreased mortality at two time points. The favorable effects of hydroxychloroquine on organ damage accrual and survival have been observed from longitudinal cohort studies [17,18]. On the other hand, the role of other immunosuppressives, especially cyclophosphamide, on the prognosis of SLE remains controversy. Cyclophosphamide is usually given together with high dose steroids for the treatment of severe and/or life threatening SLE manifestations. Evidences have suggested that the risk of end-stage renal disease in SLE patients decreased after 1980, which was coincided with increased use of cyclophosphamide [19]. However, exposure to cyclophosphamide was also shown to be independently associated with worse damage outcome [20]. It may help explain why cyclophosphamide is only beneficial for short-term survival in our cohort.

There are a number of limitations to the current study. First, over 1/3 of the patients in our database lost to contact in the past 5 years, which may cause bias in the assessment of long-term mortality. With rapid development of the economy during the past few decades, China has more and more mobile population, making it difficult to communicate people through conventional tools. Second, despite efforts to maximize data collection, there may be some important factors left out. Third, we were unable to calculate the cumulative doses of immunosuppressive medications given and this may have a bearing on damage accrual and infection related deaths. In addition, the longest follow-up period in this cohort is 15 years. For a complete understanding of long-term outcome in SLE patients, further observation is still needed.

In summary, this study shows that SLE patients had high risk of death within 1 year after the first hospitalization, especially for those with neuropsychiatric, cardiopulmonary involvements and renal insufficiency. Early and effective intervention with the use of anti-malarial drugs may be helpful to improve the prognosis of these patients.

## Supporting Information

S1 Table Division of SDI entries according to their relation to steroids treatment. (DOC)

S2 Table Causes of death in neuropsychiatric and cardiopulmonary aspects. (DOC)

S3 Table Factors associated with anti-Sm antibody. (DOC)
