# Supplementary material for: Prognosis for Hospitalized Patients with Systemic Lupus Erythematosus in China: 5-Year Update of the Jiangsu Cohort
Source: PLoS One. 2016 Dec 28;11(12):e0168619. doi: 10.1371/journal.pone.0168619 (PMC5193352; doi:10.1371/journal.pone.0168619)
Supplement: S1 Table — (DOC) [file pone.0168619.s001.doc]

**S1 Table. Division of SDI entries according to their relation to steroids treatment.**

|  | Alive | Deceased within one year | Deceased after one year |
| --- | --- | --- | --- |
| Definitely or possibly related (%) | 76 (6.7) | 14 (13.3)* | 10 (8.8) |
| Ocular | 3 | 0 | 1 |
| Musculoskeletal | 22 | 4 | 3 |
| Cardiovascular | 14 | 4 | 1 |
| Peripheral vascular disease | 7 | 0 | 0 |
| Neuropsychiatric | 19 | 4 | 5 |
| Diabetes | 11 | 2 | 0 |
| Independent of steroids (%) | 161 (14.2) | 11 (10.5) | 14 (12.3) |
| Renal/pulmonary | 119 | 7 | 12 |
| Gastrointestinal | 6 | 0 | 0 |
| Skin | 25 | 4 | 1 |
| Gonadal/malignancy | 11 | 0 | 1 |

*p < 0.05 vs. patients alive.
