# Supplementary material for: Prognosis for Hospitalized Patients with Systemic Lupus Erythematosus in China: 5-Year Update of the Jiangsu Cohort
Source: PLoS One. 2016 Dec 28;11(12):e0168619. doi: 10.1371/journal.pone.0168619 (PMC5193352; doi:10.1371/journal.pone.0168619)
Supplement: S2 Table — (DOC) [file pone.0168619.s002.doc]

**S2 Table. Causes of death in neuropsychiatric and cardiopulmonary aspects.**

| Cause of death | Deceased within one year  (n) | Deceased after one year  (n) |
| --- | --- | --- |
| Neuropsychiatric |  |  |
| Neuropsychiatric lupus | 17 | 8 |
| Intracranial hemorrhage | 5 | 2 |
| Cerebral hernia | 3 | 0 |
| Cardiopulmonary |  |  |
| Pulmonary arterial hypertension | 3 | 7 |
| Pulmonary involvement | 0 | 5 |
| Myocardial damage | 1 | 1 |
| Sudden death | 0 | 2 |
| Aortic dissection | 0 | 1 |
