# Supplementary material for: Prognosis for Hospitalized Patients with Systemic Lupus Erythematosus in China: 5-Year Update of the Jiangsu Cohort
Source: PLoS One. 2016 Dec 28;11(12):e0168619. doi: 10.1371/journal.pone.0168619 (PMC5193352; doi:10.1371/journal.pone.0168619)
Supplement: S3 Table — (DOC) [file pone.0168619.s003.doc]

**S3 Table. Factors associated with anti-Sm antibody (univariate logistic regression).**

| Factors | Deceased within one year | | | Deceased after one year | | |
| --- | --- | --- | --- | --- | --- | --- |
|  | OR | 95%CI | p | OR | 95%CI | p |
| Age | 0.70 | 0.54-0.91 | 0.008 | 0.72 | 0.55-0.93 | 0.014 |
| Gender | 1.69 | 1.08-2.65 | 0.021 | 1.61 | 1.01-2.55 | 0.045 |
| Duration | 0.76 | 0.59-0.98 | 0.034 | 0.71 | 0.54-0.91 | 0.008 |
| SLEDAI | 1.28 | 0.99-1.65 | 0.057 | 1.29 | 1.00-1.66 | 0.050 |
| Mucocutaneous | 1.03 | 0.79-1.35 | >0.10 | 0.98 | 0.75-1.29 | >0.10 |
| Musculoskeletal | 0.96 | 0.75-1.24 | >0.10 | 0.95 | 0.74-1.22 | >0.10 |
| Neuropsychiatric | 1.32 | 0.81-2.15 | >0.10 | 1.32 | 0.79-2.20 | >0.10 |
| Cardiopulmonary | 1.25 | 0.92-1.70 | >0.10 | 1.26 | 0.92-1.72 | >0.10 |
| Gastrointestinal | 1.18 | 0.88-1.60 | >0.10 | 1.27 | 0.94-1.72 | >0.10 |
| Renal | 0.89 | 0.69-1.14 | >0.10 | 0.82 | 0.64-1.06 | >0.10 |
| Hematologic | 0.96 | 0.74-1.23 | >0.10 | 0.96 | 0.75-1.24 | >0.10 |
| C3 | 2.03 | 1.46-2.81 | 0.000 | 1.84 | 1.34-2.55 | 0.000 |
| C4 | 1.74 | 1.21-2.51 | 0.003 | 1.63 | 1.14-2.34 | 0.008 |
| RF | 1.62 | 1.16-2.26 | 0.004 | 1.71 | 1.22-2.39 | 0.002 |
